# Machine Learning Models Versus the National Early Warning Score System for Predicting Deterioration: Retrospective Cohort Study in the United Arab Emirates

**DOI:** 10.2196/45257

**Published:** 2023-11-06

**Authors:** Hazem Lashen, Terrence Lee St John, Y Zaki Almallah, Madhu Sasidhar, Farah E Shamout

**Affiliations:** 1 Engineering Division New York University Abu Dhabi Abu Dhabi United Arab Emirates; 2 Cleveland Clinic Abu Dhabi Abu Dhabi United Arab Emirates; 3 Cleveland Clinic Tradition Hospital Port St. Lucie, FL United States

**Keywords:** machine learning, early warning score system, clinical deterioration, early warning, score system, cohort, real-world data, neural network, predict, deterioration

## Abstract

**Background:**

Early warning score systems are widely used for identifying patients who are at the highest risk of deterioration to assist clinical decision-making. This could facilitate early intervention and consequently improve patient outcomes; for example, the National Early Warning Score (NEWS) system, which is recommended by the Royal College of Physicians in the United Kingdom, uses predefined alerting thresholds to assign scores to patients based on their vital signs. However, there is limited evidence of the reliability of such scores across patient cohorts in the United Arab Emirates.

**Objective:**

Our aim in this study was to propose a data-driven model that accurately predicts in-hospital deterioration in an inpatient cohort in the United Arab Emirates.

**Methods:**

We conducted a retrospective cohort study using a real-world data set that consisted of 16,901 unique patients associated with 26,073 inpatient emergency encounters and 951,591 observation sets collected between April 2015 and August 2021 at a large multispecialty hospital in Abu Dhabi, United Arab Emirates. The observation sets included routine measurements of heart rate, respiratory rate, systolic blood pressure, level of consciousness, temperature, and oxygen saturation, as well as whether the patient was receiving supplementary oxygen. We divided the data set of 16,901 unique patients into training, validation, and test sets consisting of 11,830 (70%; 18,319/26,073, 70.26% emergency encounters), 3397 (20.1%; 5206/26,073, 19.97% emergency encounters), and 1674 (9.9%; 2548/26,073, 9.77% emergency encounters) patients, respectively. We defined an adverse event as the occurrence of admission to the intensive care unit, mortality, or both if the patient was admitted to the intensive care unit first. On the basis of 7 routine vital signs measurements, we assessed the performance of the NEWS system in detecting deterioration within 24 hours using the area under the receiver operating characteristic curve (AUROC). We also developed and evaluated several machine learning models, including logistic regression, a gradient-boosting model, and a feed-forward neural network.

**Results:**

In a holdout test set of 2548 encounters with 95,755 observation sets, the NEWS system achieved an overall AUROC value of 0.682 (95% CI 0.673-0.690). In comparison, the best-performing machine learning models, which were the gradient-boosting model and the neural network, achieved AUROC values of 0.778 (95% CI 0.770-0.785) and 0.756 (95% CI 0.749-0.764), respectively. Our interpretability results highlight the importance of temperature and respiratory rate in predicting patient deterioration.

**Conclusions:**

Although traditional early warning score systems are the dominant form of deterioration prediction models in clinical practice today, we strongly recommend the development and use of cohort-specific machine learning models as an alternative. This is especially important in external patient cohorts that were unseen during model development.

## Introduction

### Background

Early warning score (EWS) systems are a staple of modern clinical practice because they provide a standardized method for detecting in-hospital patient deterioration. Several other systems have been introduced with the advent of computerized medical records [1,2], such as the Modified Early Warning Score system [3] and the National Early Warning Score (NEWS) system [4], which is recommended by the Royal College of Physicians in the United Kingdom.

Such systems assign an overall aggregate score to the patient to indicate their overall risk of deterioration, based on a predetermined set of alerting ranges [5]; for example, the alerting thresholds of the NEWS system are shown in Table 1. Later work introduced EWS systems tailored for specific patient subgroups, such as for pediatrics [6] or cardiovascular-related deterioration [7]. The main strengths of EWS systems are that they are simple, easy to use, and highly interpretable [8,9], which facilitates their use in hospitals, including those with limited resources [10,11].

**Table 1:** Summary of the National Early Warning Score system, with the thresholds of the system outlined. For a given set of vital signs measurements, each variable is compared against its respective threshold and assigned a score accordingly. The patient’s overall score is the summation of scores assigned to all variables.

Vital sign	Score						
	3	2	1	0	1	2	3
Heart rate (beats/min)	≤40	N/A^a^	41-50	51-90	91-110	111-130	≥131
Oxygen saturation (%)	≤91	92-93	94-95	≥96	N/A	N/A	N/A
Temperature (°C)	≤35	N/A	35.1-36.0	36.1-38.0	38.1-39.0	≥39.1	N/A
Systolic blood pressure (mm Hg)	≤90	91-100	101-110	111-219	N/A	N/A	≥220
Respiratory rate (breaths/min)	≤8	N/A	9-11	12-20	N/A	21-24	≥25
Level of consciousness	N/A	N/A	N/A	Alert	N/A	N/A	Voice, pain, or unresponsive
Supplementary oxygen	N/A	Yes	N/A	No	N/A	N/A	N/A

^a^N/A: not applicable.

Despite their ubiquity, EWS systems also have limitations. Many of the alerting thresholds are defined in a heuristic manner with respect to a specific deterioration timeline, which makes it increasingly difficult to modify the thresholds for cohorts with significantly different characteristics or demographics than those relied upon during model development, as witnessed during the COVID-19 pandemic [12-14]. In addition, EWS systems do not capture any relationships between the input variables and commonly treat them equally, despite some being more indicative of deterioration than others [1]. However, because of their simplicity, they have been widely deployed in hospitals around the world.

In recent years, machine learning (ML) techniques have gained popularity in the development of deterioration prediction models [15-18] by treating the problem as a binary classification task [19-21]. Such approaches range from gradient-boosted trees [12,22], which consist of an ensemble of tree models, to neural networks (NNs) [21,23] and have been used in different scenarios where deterioration prediction is needed [24-26]. Although ML models have been shown to outperform traditional EWS systems [19,27], especially during the COVID-19 pandemic [28,29], one of their main limitations is the lack of interpretability compared with traditional EWS systems [30].

### Objectives

Our aim in this study was to propose a data-driven model that predicts patient deterioration with high accuracy in an inpatient cohort in Abu Dhabi, United Arab Emirates. To this end, we assessed and compared the performance of the NEWS system with that of 3 ML models, namely logistic regression (LR), gradient-boosted trees, and NNs, and developed and evaluated the models using a real-world data set collected at a multispecialty hospital in Abu Dhabi. We also used Shapley additive explanations (SHAP) analysis as a way to interpret the predictions of the ML models.

## Methods

This study is reported in accordance with the Transparent Reporting of a Multivariable Prediction Model for Individual Prognosis or Diagnosis (TRIPOD) guidelines [31]. The TRIPOD checklist can be found in Multimedia Appendix 1.

### Ethics Approval

The study received approval from the research ethics committees at Cleveland Clinic Abu Dhabi (A-2020-102) and New York University Abu Dhabi (HRPP-2020-55).

### Data Set

We obtained a data set collected between April 2015 and August 2021 at the multispecialty facility Cleveland Clinic Abu Dhabi in Abu Dhabi, United Arab Emirates. The data set included patient demographics; vital signs measurements; and time stamps relating to admission to the intensive care unit (ICU) and mortality, which are the adverse events of interest in this study.

**Figure 1:**
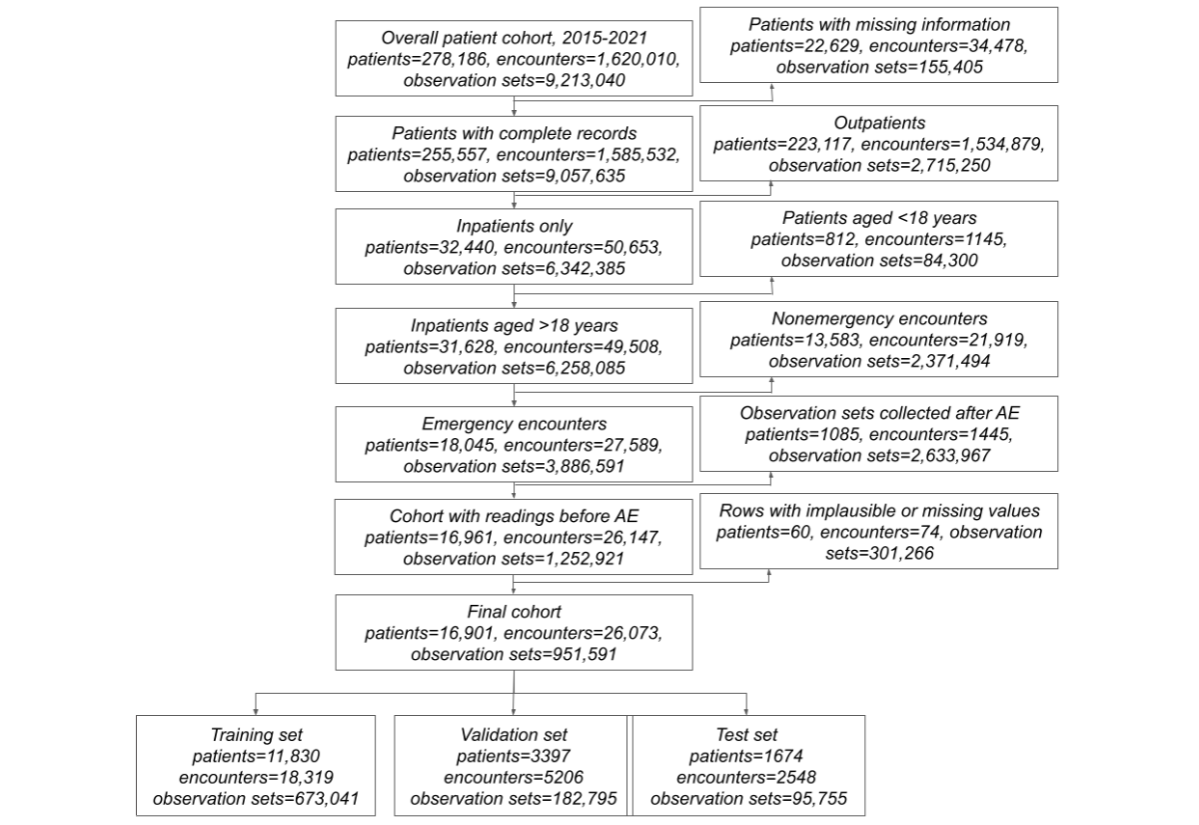
Application of the inclusion and exclusion criteria. We illustrate here the results of applying the inclusion and exclusion criteria, where p, e, and n represent the number of patients, encounters, and observation sets, respectively. We first excluded patients with missing information, such as age or patient identifiers. We included inpatient encounters of adult patients (aged >18 years) and excluded nonemergency encounters. Finally, we excluded observation sets recorded after an adverse event (AE) had occurred as well as observation sets with ≥1 implausible observations.

**Figure 2:**
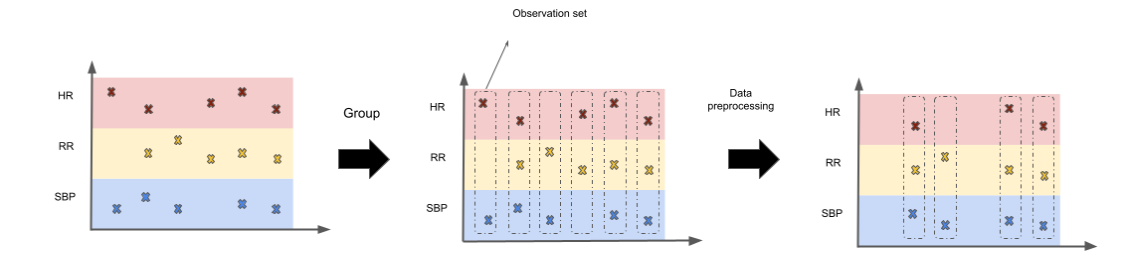
Definition of observation sets. We illustrate here a simplified version of how we defined observation sets using 3 vital signs only: heart rate (HR), respiratory rate (RR), and systolic blood pressure (SBP). Vital signs taken at the same time were grouped together for the same patient encounter into observation sets. We subsequently applied our inclusion and exclusion criteria to select the relevant patient encounters and associated observation sets.

We defined the inclusion and exclusion criteria following the standard in previous work for the development of EWS systems [2] (Figure 1). First, we grouped a set of vital signs measurements to represent a single observation set if they had been recorded within the same patient encounter and shared the same time of measurement. We excluded any patients with missing identifiers or necessary information such as records pertaining to whether the patient was alive at the time of discharge, patient age, and patient or encounter identifiers, as well as time stamps of vital signs measurements. We included inpatient admissions and excluded encounters of patients aged <18 years at the time of admission. We only included emergency encounters and excluded other types of admissions. Within each encounter, we dropped any vital signs measurements recorded after the occurrence of an adverse event, which is essentially admission to the ICU. We excluded any observation sets that contained ≥1 implausible observations or >2 missing vital signs measurements. An illustration of our data set processing pipeline is shown in Figure 2. The plausible ranges used are presented in Table S1 in Multimedia Appendix 2.

Finally, we split the data set randomly into training, validation, and test sets in a ratio of 7:2:1, respectively. This split was carried out on a patient level such that all examples belonging to a single patient were assigned to a single split only. We split the data randomly because we assumed that most of the patients admitted in 2020 and 2021 were patients with COVID-19 infection (the COVID-19 outbreak began approximately in March 2020 in the United Arab Emirates); therefore, we were interested in assessing the average performance of the models over time. We conducted a secondary analysis where we split the data based on time to understand the impact of a temporal split.

### Input Features

We extracted 7 vital signs variables that are used in the NEWS system. These included heart rate; respiratory rate; temperature; systolic blood pressure; oxygen saturation; level of consciousness indicated by the alert, voice, pain, or unresponsive score; and whether a patient was receiving supplementary oxygen. To derive the supplementary oxygen variable, we relied on the patient’s fraction of inspired oxygen reading. We assumed that any fraction of inspired oxygen measurement of >21% indicated that the patient was receiving supplementary oxygen [32]. For level of consciousness, we used a provided binary feature in the data set that follows the scoring of the NEWS system (0 if a patient is alert and 3 otherwise). We applied mean imputation to all features, except for supplementary oxygen and level of consciousness, where a missing value was treated as not receiving supplementary oxygen and alert, respectively. We treated vital signs measurements recorded at the same time as a single observation set (Figure 2), meaning that each encounter (patient stay) contained multiple observation sets recorded at various times during the patient stay.

### Outcome Definition

We defined the composite outcome of admission to the ICU and mortality as a deterioration (adverse) event. In cases of multiple adverse events, we considered the time of the first occurring event. For a given observation set, we generated binary ground-truth labels based on whether an adverse event occurred within a certain time window from the measurement time of the respective observation set. If it did indeed occur within the time window, we set the label as 1 (positive label); otherwise, we set it as 0 (negative label). To evaluate the performance of the models over different time windows, we considered 4 different values: 6, 12, 24, and 36 hours. We note that 24 hours is the standard window of evaluation in the existing literature [33].

### Prediction Models

We developed several prediction models (refer to the following subsections) based on prevalent ML techniques. All models, except for the NEWS system, are fitted on the training set and optimized via hyperparameter tuning on the validation set, with final results being reported on the test set.

#### NEWS System

The NEWS system [4] was developed by the Royal College of Physicians to provide a standardized EWS system to easily and quickly identify patients at high risk of deterioration. The NEWS system assigns a score to 7 vital signs measurements based on predetermined alerting thresholds (Table 1). The higher the final score, the greater the risk of deterioration. For each observation set, we calculated the total score based on the scores assigned to each vital sign. We then normalized each score by dividing it by the maximum possible NEWS score, which is 20, to compute performance metrics.

#### Gradient-Boosting Model

We developed a gradient-boosting model, extreme gradient boosting (XGBoost) [22,34], that uses an ensemble of decision trees. We implemented this model using the *XGBoost* package [34].

#### LR Model

LR [35] is a simple statistical method that assumes a linear combination of the input variables and uses a sigmoid activation to compute predictions in the range between 0 and 1. We implemented this model using the *scikit learn* package [36].

#### NN Model

We implemented a feed-forward NN [37] consisting of 10 linear layers with scaled exponential linear unit activation function [38], followed by batch normalization to reduce overfitting. The outputs of the final layer are fed to a sigmoid activation function, which outputs predictions in the range between 0 and 1. For this model, we applied min-max normalization to the input features first, whereby the minimum and maximum values were defined using the training set for all data splits. We implemented this baseline using the PyTorch framework [39].

### Evaluation Metrics

We evaluated all models using 2 main evaluation metrics: the area under the receiver operating characteristic curve (AUROC) and the area under the precision-recall curve (AUPRC). Both metrics are represented as a single number between 0 and 1 to summarize the performance of a binary classifier. The receiver operating characteristic curve plots the true positive rate against the false positive rate at different classification thresholds and indicates the model’s ability to discriminate between positive and negative classes. The precision-recall curve plots precision against recall at different classification thresholds and gives an indication of the model’s average precision. The baseline performance of a random classifier is equivalent to 0.5 for the AUROC and to the ratio of positive samples to the total number of samples for the AUPRC. We computed 95% CIs for the AUROC and AUPRC metrics of all models through bootstrapping with 1000 runs [40].

In addition, we compared the difference in performance, in terms of the AUROC and AUPRC values, between each ML model and the NEWS system. We report the difference and its 95% CIs using bootstrapping with 1000 runs. We computed *P* values for each comparison using the 1-tailed permutation test with 10,000 iterations [41]. All results are reported on the test set.

### Model Selection

To develop the ML models, we used random hyperparameter search [42] to select the best hyperparameters using the validation set (XGBoost, LR, and NN). We have summarized the sampling ranges of the hyperparameters in Table S2 in Multimedia Appendix 2. We ran each model 10 times with hyperparameters selected randomly from predetermined ranges. We then selected the model with the hyperparameters that achieved the best performance on the validation set in terms of the AUPRC value because it is considered a more informative metric owing to class imbalance [43,44]. We trained the NN models for 250 epochs. We report the performance on the test set for the selected best models.

### Model Interpretability

We used the open-source SHAP package [45] to analyze feature importance using SHAP values for the best-performing model in terms of the overall AUROC. We calculated the SHAP value for each feature such that the magnitude of the SHAP value indicates greater importance for the model’s prediction, and we present the average of the absolute SHAP values for each of the 7 input features in the test set. We also present the SHAP plots for the observation sets with the highest and lowest prediction scores in the best-performing model. In addition, for each input feature, we plotted the SHAP partial dependence plot, and calculated the Pearson correlation coefficient and the Spearman rank correlation coefficient between the feature values and their respective sets of SHAP values. The partial dependence plots show the relationship between the average SHAP value and each possible vital signs measurement, whereas the coefficients indicate the overall correlation between the SHAP values and the input feature values. We also included the LR coefficients and odds ratios as a comparison point owing to the simplicity of the LR model and the significance of the coefficients in summarizing the effect of each feature on the overall prediction of the model compared with the SHAP values.

## Results

### Patient Cohort

We have summarized the results of applying the inclusion and exclusion criteria in Figure 1. Our data set comprised 1,620,010 encounters from 278,186 patients, yielding a total of 9,213,040 observation sets. Of the 278,186 patients, 255,557 (91.87%) had complete identifying information recorded in the data set, leading to the exclusion of the rest (22,629/278,186, 8.13%), leaving 97.87% (1,585,532/1,620,010) of the encounters and 98.31% (9,057,635/9,213,040) of the observation sets. Our study specifically targets inpatients; therefore, of the 255,557 patients, after excluding 223,117 (87.31%) outpatient encounters, 32,440 (12.69%) remained. Of these 32,440 patients, 31,628 (96.39%) were aged >18 years and thus eligible for inclusion (6,258,085/9,057,635, 69.09% observation sets recorded within 49,508/1,585,562, 3.12% encounters). Furthermore, we included only emergency encounters; thus, of the 31,268 patients, 18,045 (57.71%) were included (3,886,591/6,258,085, 62.11% observation sets recorded within 27,589/49,508, 55.73% encounters). We then excluded any observation sets that occurred after an adverse event, which meant that, of the 3,886,591 observation sets, 1,252,921 (32.24%) remained. Finally, of the 1,252,921 observation sets, we removed 301,266 (24.05%) that contained implausible readings for their respective vital signs, leaving 951,655 (75.95%) observation sets. Thus, of the 18,045 patients, 16,901 (93.66%) remained in the final cohort (associated with 26,073/27,589, 94.51% encounters recorded between April 2015 and August 2021). We divided the data set of 16,901 patients as follows: training set: 11,830 (70%; 18,319/26,073, 70.26% encounters), validation set: 3397 (20.1%; 5206/26,073, 19.97% encounters), and test set: 1674 (9.9%; 2548/26,073, 9.77% encounters).

We provide a summary of the cohort’s characteristics, distributions of vital signs measurements, and occurrences of adverse events in Table 2. We observed an average age of 55.3 (SD 19.3), 54.9 (SD 18.7), and 53.6 (SD 19.1) years across the training, validation, and test splits, respectively. We observed a higher proportion of male patients than female patients across all splits, with the training set comprising 59.64% (7056/11,830) male patients and the validation and testing sets comprising 60.17% (2044/3397) and 58.06% (972/1674) of male patients, respectively.

**Table 2:** Patient cohort summary. We provide a summary of the patient cohort characteristics across the training, validation, and test sets. This includes the patient demographics, distributions of input features, and the prevalence of the deterioration labels across the different time windows.

Characteristic						Training set		Validation set		Test set
**Cohort demographics**										
	**Patients (n=16,901), n (%)**					11,830 (70)		3397 (20.1)		1674 (9.9)
			Male patients^a^			7056 (59.6)		2044 (60.2)		972 (58.1)
	**Encounters (n=26,073), n (%)**					18,319 (70.3)		5206 (20)		2548 (9.8)
		**Age group (years), mean (SD)**				55.3 (19.3)		54.9 (18.7)		53.6 (19.1)
				<40, n (%)^b^	4843 (26.4)		1371 (26.3)		672 (26.4)	
				40-59, n (%)^b^	5313 (29)		1506 (28.9)		728 (28.6)	
				≥60, n (%)^b^	8163 (44.6)		2329 (44.7)		1148 (45.1)	
		Encounters with composite outcome, n (%)^b^				3979 (21.7)		1144 (22)		549 (21.5)
		Encounters during the COVID-19 pandemic, n (%)^b^				5594 (30.5)		1657 (31.8)		836 (32.8)
**Observation sets** **(n=951,591), n (%)**						673,041 (70.7)		182,795 (19.2)		95,755 (10.1)
	Heart rate (beats/min), mean (SD; IQR)					78 (15.9; 68-90)		78 (16.3; 68-89)		79 (15.8; 70-89)
	Respiratory rate (breaths/min), mean (SD; IQR)					18 (2.8; 18-20)		18 (2.9; 18-20)		18 (2.9; 18-20)
	Systolic blood pressure (mm Hg), mean (SD; IQR)					122 (20.9; 109-137)		124 (21.6; 110-139)		122 (20.2; 109-136)
	Temperature (°C), mean (SD; IQR)					36.7 (0.4; 36.5-36.9)		36.7 (0.5; 36.5-36.9)		36.7 (0.4; 36.5-36.9)
	Oxygen saturation (%), (SD; IQR)					99 (2.0; 97-100)		99 (2.0; 97-100)		99 (2.1; 97-100)
	**Level of consciousness, n (%)^c^**									
		Alert				537,853 (98.1)		144,014 (97.9)		76,376 (97.5)
		Voice, pain, or unresponsive				10,685 (1.9)		3086 (2.1)		1940 (2.5)
	**Supplementary oxygen, n (%)^d^**									
		Provided				15,201 (2.3)		3332 (1.8)		2086 (2.2)
		Not provided				657,840 (97.7)		179,463 (98.2)		93,669 (97.8)
	**Deterioration, n**									
		**Within 36 hours**				36,760		10,681		5373
				Death	1100		266		154	
				ICU^e^ admission	36,255		10,571		5319	
		**Within** **24 hours**				31,431		9306		4556
				Death	715		127		71	
				ICU admission	31,088		9233		4521	
		**Within** **12 hours**				25,382		7633		3658
				Death	358		70		21	
				ICU admission	25,199		7589		3643	
		**Within** **6 hours**				21,332		6476		2987
				Death	166		37		9	
				ICU admission	21,227		6452		2979	

^a^Training set: n=11,830; validation set: n=3397; test set: n=1674.

^b^Training set: n=18,319; validation set: n=5206; test set: n=2548.

^c^Training set: n=548,538; validation set: n=147,100; test set: n=78,316.

^d^Training set: n=673,041; validation set: n=182,795; test set: n=95,755.

^e^ICU: intensive care unit.

### Performance Compared With the NEWS System

We summarize the performances of the ML models and NEWS system in Table 3 for deterioration within 24 hours in terms of AUROC and AUPRC values. We note that the NN and XGBoost models achieved the best performance. The XGBoost model achieved an AUROC value of 0.778 (95% CI 0.770-0.785) across the entire test set. The NEWS system achieved an AUROC value of 0.682 (95% CI 0.673-0.690), which means that the XGBoost model achieved an improvement of 0.096 (95% CI 0.088-0.103; *P*<.001). In terms of the AUPRC values, compared with the NEWS system, the XGBoost model achieved an improvement of 0.093 (95% CI 0.083-0.101; *P*<.001). The NN model achieved an AUROC value of 0.756 (95% CI 0.749-0.764) and an AUPRC value of 0.222 (95% CI 0.211-0.235), leading to improvements of 0.074 (95% CI 0.067-0.081; *P*<.001) and 0.061 (95% CI 0.049-0.073; *P*<.001) in AUROC and AUPRC values, respectively, compared with the NEWS system. The LR model did not perform better than the NEWS system in terms of AUROC values, and it achieved slightly better performance in terms of AUPRC values.

**Figure 3:**
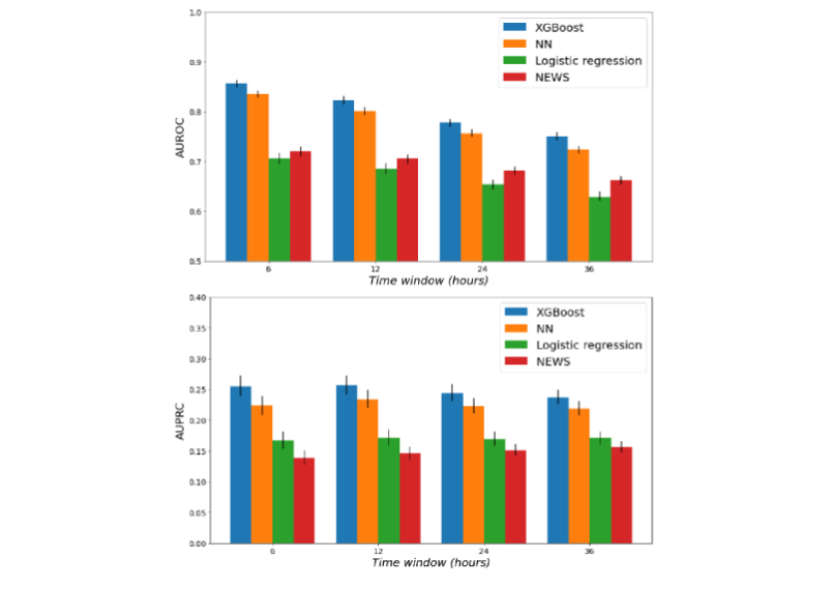
Performance of the models on the overall test set across the different prediction time windows. We evaluated the performance of each model for deterioration prediction within 6, 12, 24, and 36 hours. AUPRC: area under the precision-recall curve; AUROC: area under the receiver operating characteristic curve; NEWS: National Early Warning Score; NN: neural network; XGBoost: extreme gradient boosting.

In Figure 3, we show the AUROC and AUPRC results for all models on the test set when varying the lengths of the prediction time window as follows: 6, 12, 24, and 36 hours. We noted that the XGBoost model and the NN model performed best across all time windows, with a better performance by the XGBoost model in terms of both AUROC and AUPRC values across all time windows. We also noted a comparable performance between the NEWS system and the LR model, with the NEWS system achieving a superior AUROC value and the LR model achieving a better AUPRC value. In addition, the performance of all models decreased as the prediction time window increased. This likely indicates that the difficulty of the task increases as the adverse events occur further away in time.

**Table 3:** Model performance across different subgroups. We report performances in terms of area under the receiver operating characteristic curve (AUROC) and area under the precision-recall curve (AUPRC) values for deterioration within 24 hours in the test set. We also provide 95% CIs computed using bootstrapping.

Subgroup			XGBoost^a^				Logistic regression				Neural network				NEWS^b^		
			AUROC (95% CI)		AUPRC (95% CI)		AUROC (95% CI)		AUPRC (95% CI)		AUROC (95% CI)		AUPRC (95% CI)		AUROC (95% CI)		AUPRC (95% CI)
All patients			*0.778*^c^ (0.770-0.785)		*0.244* (0.231-0.258)		0.654 (0.644-0.663)		0.169 (0.158-0.181)		0.756 (0.749-0.764)		0.222 (0.211-0.235)		0.682 (0.673-0.690)		0.151 (0.142-0.161)
Male patients			*0.775* (0.764-0.784)		*0.274* (0.258-0.291)		0.651 (0.638-0.663)		0.208 (0.193-0.223)		0.752 (0.742-0.762)		0.253 (0.237-0.269)		0.675 (0.665-0.685)		0.176 (0.163-0.190)
Female patients			*0.785* (0.772-0.797)		*0.214* (0.194-0.236)		0.676 (0.662-0.692)		0.137 (0.123-0.155)		0.766 (0.754-0.779)		0.194 (0.176-0.216)		0.704 (0.689-0.718)		0.129 (0.116-0.145)
**Age group (years)**																	
	<40	*0.818* (0.797-0.837)		*0.222* (0.193-0.256)		0.739 (0.718-0.761)		0.153 (0.130-0.179)		0.804 (0.784-0.824)		0.213 (0.184-0.249)		0.738 (0.717-0.758)		0.120 (0.104-0.138)	
	40-59	*0.758* (0.744-0.772)		*0.251* (0.230-0.275)		0.609 (0.592-0.626)		0.159 (0.143-0.176)		0.734 (0.721-0.749)		0.226 (0.208-0.248)		0.640 (0.626-0.655)		0.149 (0.134-0.165)	
	≥60	*0.779* (0.768-0.790)		*0.258* (0.240-0.278)		0.663 (0.649-0.676)		0.196 (0.181-0.214)		0.757 (0.745-0.768)		0.235 (0.218-0.254)		0.700 (0.689-0.712)		0.177 (0.162-0.192)	

^a^XGBoost: extreme gradient boosting.

^b^NEWS: National Early Warning Score.

^c^The best results in each subgroup are italicized.

### Performance Across Different Patient Subgroups

In Table 3, we have also summarized the performance of the models across different patient subgroups within the test set. Across the male population, the XGBoost model achieved the best performance in terms of AUROC values (0.775, 95% CI 0.764-0.784), with an improvement of 0.099 (95% CI 0.090-0.109; *P*<.001) compared with the NEWS system. The XGBoost model also achieved the best performance in the female population with an AUROC value of 0.785 (95% CI 0.772-0.797) and an AUPRC value of 0.214 (95% CI 0.194-0.236), which corresponds to improvements of 0.081 (95% CI 0.070-0.092; *P*<.001) in AUROC value and 0.084 (95% CI 0.070-0.099; *P*<.001) in AUPRC value compared with the NEWS system.

In the different age subpopulations, the XGBoost model achieved the best results (AUROC 0.758-0.818), followed by the NN model (AUROC 0.721-0.760). In the population consisting of patients aged <40 years, the XGBoost model achieved the best performance in terms of AUROC value (0.818, 95% CI 0.797-0.837) and AUPRC value (0.222, 95% CI 0.193-0.256), with improvements of 0.080 (95% CI 0.064-0.097; *P*<.001) and 0.102 (95% CI 0.082-0.125; *P*<.001) in AUROC and AUPRC values, respectively, compared with the NEWS system. In the group consisting of patients aged 40-59 years, the XGBoost model achieved an AUROC value of 0.758 (95% CI 0744-0.772) and an AUPRC value of 0.251 (95% CI 0.230-0.275), with improvements of 0.118 (95% CI 0.104-0.133; *P*<.001) and 0.102 (95% CI 0.087-0.119; *P*<.001) in AUROC and AUPRC values, respectively, compared with the NEWS system.

Finally, in the group consisting of patients aged ≥60 years, the XGBoost model achieved an AUROC value of 0.779 (95% CI 0.768-0.790) and an AUPRC value of 0.258 (95% CI 0.240-0.278), with improvements of 0.078 (95% CI 0.069-0.088; *P*<.001) and 0.081 (95% CI 0.068-0.094; *P*<.001) in AUROC and AUPRC values, respectively, compared with the NEWS system.

### Performance Based on a Temporal Data Split

Given that most of the patient cohort during 2020-2021 consisted of patients with COVID-19 infection, we investigated the impact of increasing the size of the training set based on a temporal data split for deterioration within 24 hours. To do so, we defined four training sets that encompassed data collected during (1) 2016, (2) 2016-2017, (3) 2016-2018, and (4) 2016-2019. We defined a new test set that included the observation sets of all patients admitted to the hospital in 2020. We excluded any data collected during 2015 and 2021 because our data set only included a few months from both years. The new test set consisted of 517 unique patients (307/517, 59.4% male patients), with 638 encounters associated with an average age of 54.0 years and 23,227 observation sets (1208/23,227, 5.2% deterioration within 24 hours).

We summarize the results in Table 4. We observed that increasing the size of the training set yielded marginal improvements in terms of AUROC and AUPRC values across all models. The NN model saw the largest improvement in AUROC value, which increased from 0.706 (95% CI 0.688-0.722) to 0.754 (95% CI 0.739-0.769), whereas the XGBoost model saw the largest improvement in AUPRC value, which increased from 0.207 (0.187-0229) to 0.250 (95% CI 0.226-0.276).

**Table 4:** Model performance based on a temporal data split for deterioration within 24 hours. We performed a temporal data split for the training and test sets. We fixed the test set to patient encounters recorded during 2020, whereas we expanded the training set gradually to eventually include encounters recorded between 2016 and 2019. We report area under the receiver operating characteristic curve (AUROC) and area under the precision-recall curve (AUPRC) values with 95% CIs.

Training set	Deterioration within 24 h, n (%)	XGBoost^a^			Logistic regression			Neural network	
		AUROC (95% CI)	AUPRC (95% CI)	AUROC (95% CI)		AUPRC (95% CI)	AUROC (95% CI)		AUPRC (95% CI)
2016: n=68,499	2788 (4.1)	0.740 (0.724-0.754)	0.207 (0.187-0.229)	0.679 (0.660-0.696)		0.204 (0.182-0.228)	0.706 (0.688-0.722)		0.211 (0.188-0.233)
2016-2017: n=159,888	7062 (4.4)	0.763 (0.747-0.778)	0.232 (0.211-0.257)	0.687 (0.669-0.704)		0.213 (0.191-0.237)	0.744 (0.727-0.759)		0.241 (0.216-0.266)
2016-2018: n=285,733	12,352 (4.3)	0.758 (0.742-0.773)	0.242 (0.218-0.267)	0.69 (0.671-0.706)		0.216 (0.194-0.240)	0.745 (0.728-0.760)		0.237 (0.213-0.262)
2016-2019: n=431,503	19,261 (4.5)	0.778 (0.763-0.792)	0.25 (0.226-0.276)	0.688 (0.670-0.705)		0.215 (0.192-0.239)	0.754 (0.739-0.769)		0.233 (0.211-0.259)

^a^XGBoost: extreme gradient boosting.

### Interpretability Results

Table 5 shows the overall importance of each input feature in the XGBoost model predicting deterioration within 24 hours. The plots for the other time windows (6, 12, and 36 hours) are shown in Figure S1 in Multimedia Appendix 2. We observed a similar pattern across all time window values. We noted that temperature is the most important feature, followed closely by respiratory rate, systolic blood pressure, heart rate, level of consciousness, oxygen saturation, and finally provision of supplementary oxygen.

**Figure 4:**
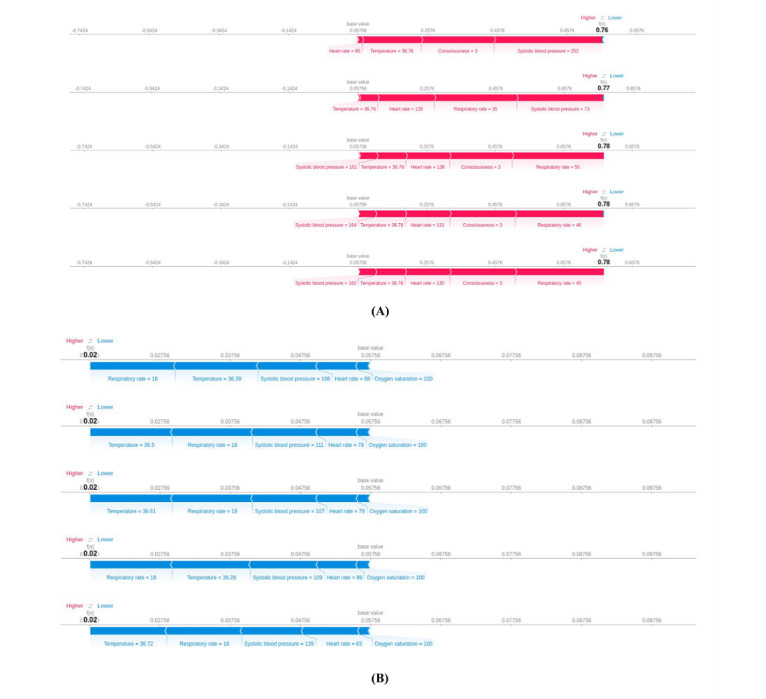
Feature importance of the highest and lowest predictions of deterioration in the test set. We present the Shapley additive explanations (SHAP) values for (A) 5 observation sets with the highest predictions of deterioration assigned by the extreme gradient boosting (XGBoost) model in the test set and (B) 5 observation sets with the lowest predictions of deterioration. We confirmed that all observation sets in (A) did indeed experience an adverse event within 24 hours, whereas all observation sets in (B) did not. Note that temperature values are displayed in degrees Fahrenheit. For a higher-resolution version of this figure, see Multimedia Appendix 3.

Figure 4 shows the SHAP values and the corresponding feature values of the observation sets that were assigned the highest and lowest predictions of deterioration. We observed that for all observation sets with the highest assigned probabilities, the factors contributing the most were high or low systolic blood pressure measurements, level of consciousness where a value of 3 indicated that the patient was unconscious, and high heart rate measurements. In the 5 observation sets with the lowest assigned probabilities, the patients displayed mostly normal vital signs measurements.

**Figure 5:**
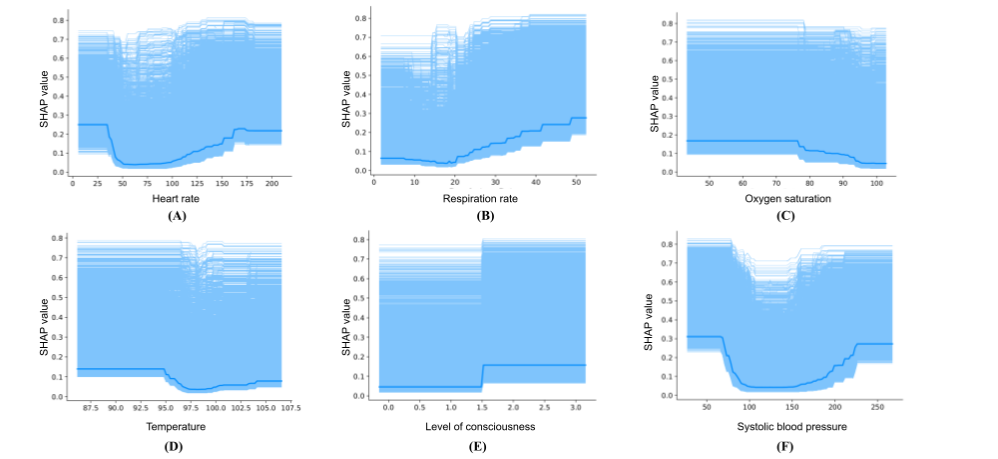
Partial dependence plots for the input features. We present the Shapley additive explanations (SHAP) partial dependence plots for six input features: (A) heart rate, (B) respiratory rate, (C) oxygen saturation, (D) temperature, (E) level of consciousness, and (F) systolic blood pressure. The partial dependence plot for supplementary oxygen is a flat line; hence, it has been omitted from the figure.

Figure 5 shows the SHAP partial dependence plots for 6 (86%) of the 7 input features. We observed that for continuous variables (eg, heart rate, respiratory rate, oxygen saturation, temperature, and systolic blood pressure), there is a range of values for which the SHAP contributions are the lowest; for example, for heart rate, the average SHAP value encounters the sharpest drop between approximately 50 and 100 beats per minute. For oxygen saturation, we observed that the SHAP values decreased as oxygen saturation increased to >80%, whereas for respiratory rate, we observed that SHAP values increased as respiratory rate increased. Level of consciousness is a binary variable, and it can be observed in Figure 5 that the average SHAP value for level of consciousness varies based on whether the patient is conscious.

Table 6 shows the Pearson correlation coefficients and Spearman rank correlation coefficients between the SHAP values and the feature values, as well as the LR coefficients and odds ratios. We observed that level of consciousness shows the highest level of correlation (Pearson correlation coefficient=0.950, Spearman rank correlation coefficient=1.000, and LR coefficient=0.532). We also noted that the LR coefficients are aligned with those of SHAP, based on the relative ranking of the features with the calculated Pearson coefficients and the LR coefficients. Temperature exhibits the lowest level of correlation, perhaps because of the complexity of the nonlinear relationship between the feature and the outcome variable.

**Table 5:** Feature importance of the extreme gradient boosting model. We present the results of our Shapley additive explanations (SHAP) analysis for the extreme gradient boosting model for the deterioration within each of our proposed time windows. We provide the mean of the absolute SHAP value for each of the 7 input features.

Vital sign	Mean of absolute SHAP value			
	36 hours	24 hours	12 hours	6 hours
Temperature	0.019	0.018	0.018	0.018
Respiratory rate	0.016	0.015	0.015	0.012
Systolic blood pressure	0.013	0.013	0.013	0.011
Heart rate	0.011	0.010	0.010	0.008
Level of consciousness	0.003	0.003	0.003	0.002
Oxygen saturation	0.003	0.003	0.003	0.002
Supplementary oxygen	0.000	0.000	0.000	0.000

**Table 6:** Feature correlation values. We summarize the Pearson correlation coefficients and Spearman rank correlation coefficients between the calculated Shapley additive explanations (SHAP) values and the respective features. For each feature, we also present the coefficients of the logistic regression model and their respective odds ratios.

	Pearson correlation coefficient	Spearman rank correlation coefficient	Logistic regression coefficient	Logistic regression odds ratio
Heart rate	0.606	0.816	0.016	1.016
Respiratory rate	0.792	0.417	0.128	1.137
Oxygen saturation	−0.713	−0.694	−0.073	0.930
Temperature	0.006	−0.068	0.001	1.002
Level of consciousness	0.950	1.000	0.532	1.702
Supplementary oxygen	0.021	0.080	0.011	1.011
Systolic blood pressure	−0.060	−0.099	0.001	1.001

## Discussion

### Principal Findings

EWS systems provide a standardized method for the detection of patient deterioration [46]. Despite the proliferation of EWS systems in electronic health record systems, they are often developed based on heuristics or data acquired from a specific patient cohort [12]. One such EWS system is the NEWS system [4], which is recommended by the Royal College of Physicians and is currently in use in some hospitals in the United Arab Emirates. In this work, we developed and evaluated data-driven deterioration prediction models using ML and real-world data collected at a local hospital. We compared the performance of the ML models with that of the NEWS system in a holdout test set consisting of 2548 encounters and 95,755 observation sets in terms of AUROC and AUPRC values.

Our study has several strengths. First, in the overall population, our results showed that the XGBoost model and the NN model achieved the best performance with improvements of 0.096 (95% CI 0.088-0.103; *P*<.001) and 0.074 (95% CI 0.067-0.081; *P*<.001), respectively, compared with the NEWS system. This is consistent with the findings of other studies, where the XGBoost model predominantly achieved the best performance compared with other models, especially with tabular input data [34,47,48]. Considering the performance improvement with respect to the NEWS system, we suggest in this case that a hospital is likely to benefit more by developing its own models using cohort-specific data, instead of relying on external models [49]. However, this requires expertise and computational resources that may not always be readily available. In addition, we showed that although the models’ performance remained stable as the training sets were expanded, and more data were collected, future work should focus on tackling distribution shifts owing to changes in practice over time or changes in patient phenotype and demographics. The discrepancy in performance across all models when using a random data split compared with a temporal split also highlights the importance of choosing training and test sets that best reflect the eligible population during model deployment and implementation.

Another strength of our study is that we assessed the performance of the models across different deterioration windows. We showed that as the prediction window increased in size, the predictive performance of all models decreased because the level of difficulty of the prediction tasks increased. This implies that, in practice, one must deploy the model that best aligns with the interventions that can be implemented. We also assessed the importance of the input features as an interpretability mechanism. In predicting deterioration within 24 hours, respiratory rate was among the top 2 most important features. This is in line with existing work that emphasizes the importance of respiratory rate as a clinical biomarker and indicator of patient status [50].

Despite the contributions of our study in proposing a new deterioration prediction model for the United Arab Emirates population, our study has some limitations. We only assessed the performance of our model using an internal test set from a single center because we did not have access to any external validation cohorts. In addition, our model relied on a small set of 7 input features, mostly vital signs, and we did not include any other variables that may be indicative of deterioration, such as laboratory test results. We performed a patient-level split across the training, validation, and test splits to avoid data leakage across the data splits. However, this could potentially bias the learning of the model owing to patients having multiple encounters or observation sets within a specific data split. On average, each unique patient had 1.6, 1.5, and 1.5 encounters in the training, validation, and test sets, respectively; therefore, we suspect low levels of bias, although this is a limitation of the training strategy. As we developed models that computed predictions every time an observation set was recorded, to mimic EWS systems in real time, we also included all observation sets of all encounters. In future work, more advanced data-split training and evaluation strategies can be investigated for encounter-level predictions with more advanced methods that consider time-series analysis.

Future work should also focus on the development of multimodal EWS systems, including imaging modalities such as chest x-ray images [51]. However, this depends on the target population of the EWS system and the availability of multimodal data. We also did not assess the performance of the latest version of the NEWS system [1,52], also referred to as NEWS2, which introduced specific alerting thresholds for patients with hypercapnic respiratory failure in a current or previous encounter, and this is an area of future work. Another area of future work with expected clinical impact would be to study how existing patient management protocols can be re-evaluated with respect to the model’s predictions and marginal risk measures computed using SHAP analysis for the input features.

### Conclusions

In conclusion, we developed and evaluated deterioration prediction models using ML and a real-world data set and compared their performance with that of the NEWS system, which is commonly used in practice. In future work, we will seek to evaluate the performance of the XGBoost model in a silent prospective validation study to verify further areas of improvement. Although we developed models specific to our patient cohort, we believe that our framework may be useful to other researchers interested in developing and evaluating deterioration prediction models.

## Appendix

Multimedia Appendix 1 Transparent Reporting of a Multivariable Prediction Model for Individual Prognosis or Diagnosis (TRIPOD) checklist.

Multimedia Appendix 2 Supplementary figures and tables.

Multimedia Appendix 3 Feature importance of the highest and lowest predictions of deterioration in the test set. We present the Shapley additive explanations (SHAP) values for (A) 5 observation sets with the highest predictions of deterioration assigned by the extreme gradient boosting (XGBoost) model in the test set and (B) 5 observation sets with the lowest predictions of deterioration. We confirmed that all observation sets in (A) did indeed experience an adverse event within 24 hours, whereas all observation sets in (B) did not. Note that temperature values are displayed in degrees Fahrenheit.

Multimedia Appendix 4 Synthetic data that are randomly generated to closely resemble the original data set used in this study.

## Abbreviations

AUPRC: area under the precision-recall curve
AUROC: area under the receiver operating characteristic curve
EWS: early warning score
ICU: intensive care unit
LR: logistic regression
ML: machine learning
NEWS: National Early Warning Score
NN: neural network
SHAP: Shapley additive explanations
TRIPOD: Transparent Reporting of a Multivariable Prediction Model for Individual Prognosis or Diagnosis
XGBoost: extreme gradient boosting

## Data Availability

Synthetic data that are randomly generated to closely resemble the original data set used in this study is available in Multimedia Appendix 4
