# Supplementary figures and images for: Machine Learning Models Versus the National Early Warning Score System for Predicting Deterioration: Retrospective Cohort Study in the United Arab Emirates
Source: JMIR AI. 2023 Nov 6;2:e45257. doi: 10.2196/45257 (PMC11041421; doi:10.2196/45257)

**TRIPOD Statement**


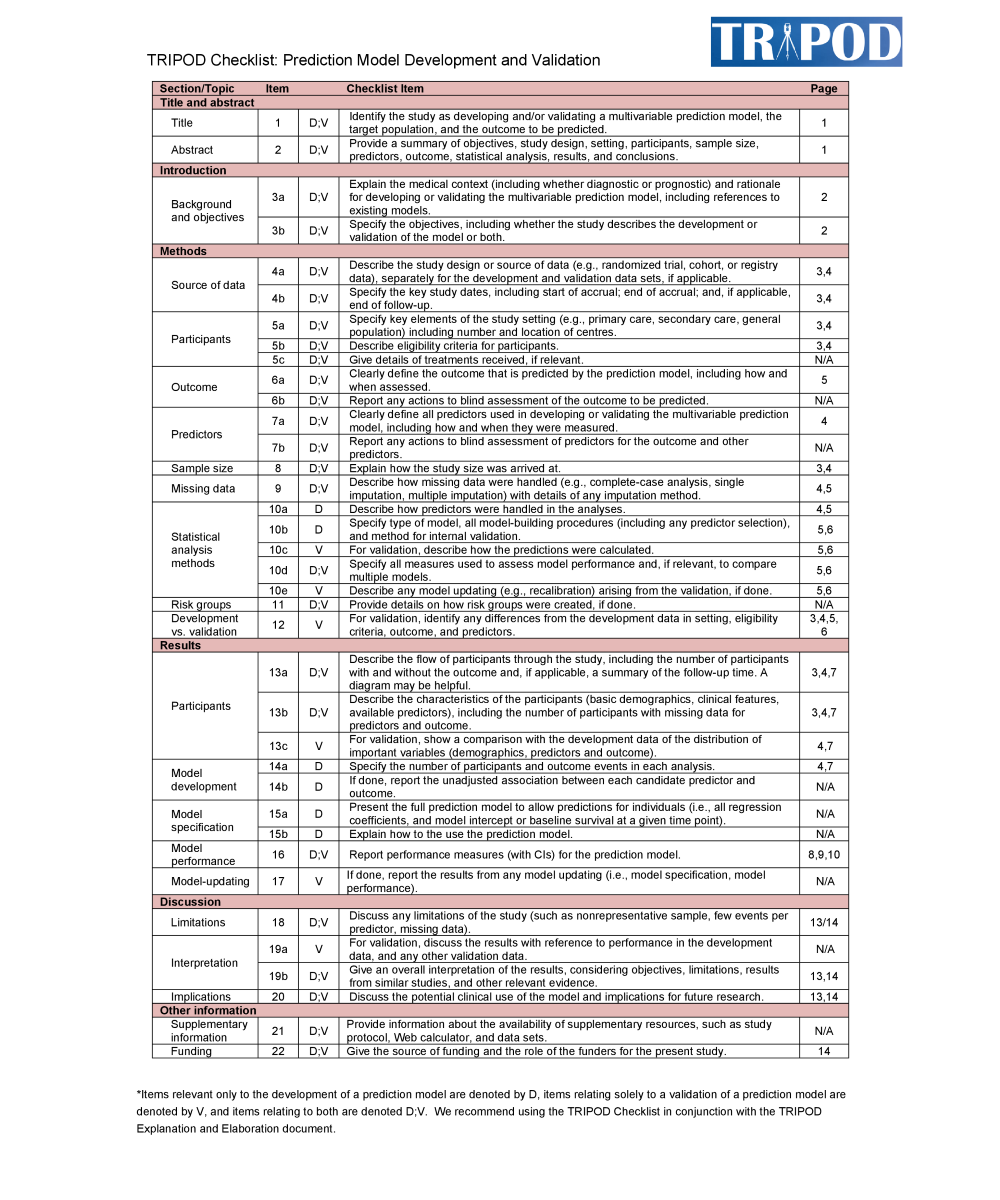

Supplement: Multimedia Appendix 1 [file ai_v2i1e45257_app1.docx]

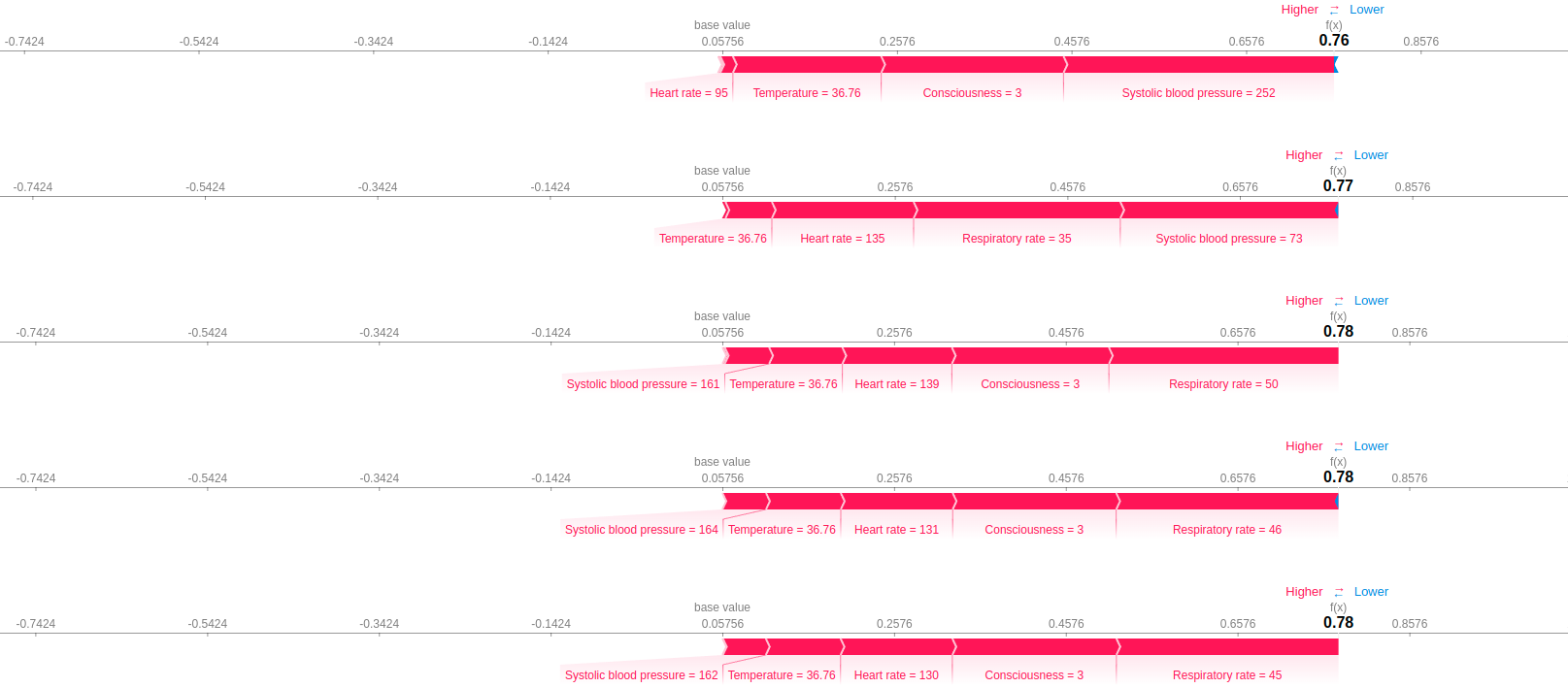
**(A)**

**
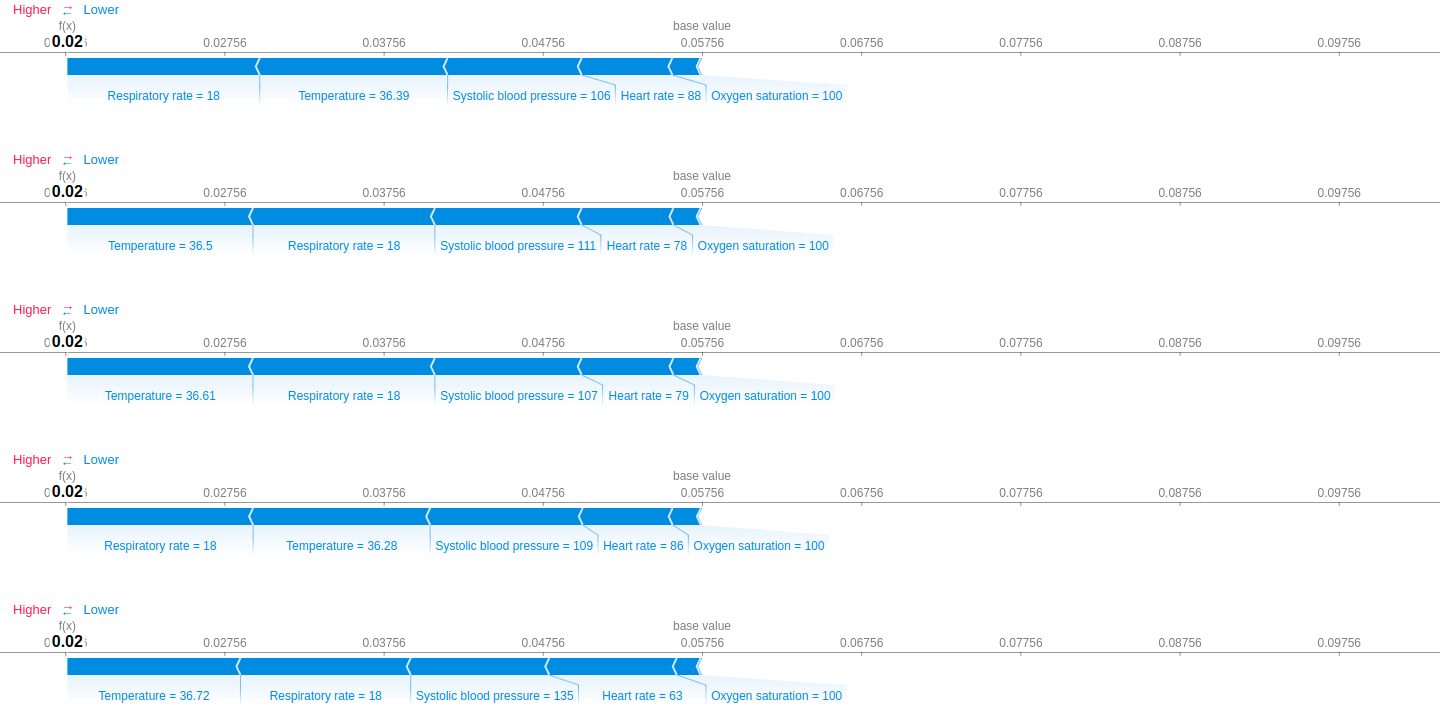
(B)**

Supplement: Multimedia Appendix 3 [file ai_v2i1e45257_app3.docx]
