# Supplementary material for: Machine Learning Models Versus the National Early Warning Score System for Predicting Deterioration: Retrospective Cohort Study in the United Arab Emirates
Source: JMIR AI. 2023 Nov 6;2:e45257. doi: 10.2196/45257 (PMC11041421; doi:10.2196/45257)
Supplement: Multimedia Appendix 2 [file ai_v2i1e45257_app2.docx]

# **Implementation details**

**Plausible Ranges**

We report the plausible ranges used in our inclusion exclusion criteria in Table S1.

**Table S1. Plausible ranges used for each variable reported in the specified unit of measurement.** Observation sets in the dataset that include measurements outside of these specified ranges are removed as specified by the inclusion and exclusion criteria outlined in Section 2.

| **Physiological variable (units)** | **Minimum** | **Maximum** |
| --- | --- | --- |
| Heart Rate (beats per minute) | 15 | 250 |
| Respiratory Rate (breaths per minute) | 3 | 50 |
| Systolic Blood Pressure (mmHg) | 30 | 300 |
| Temperature (°C) | 28 | 45 |
| Oxygen Saturation (%) | 40 | 100 |

**SHAP Values for other values of *N***

We report the SHAP values for *N* =6*,* 12, and 36 hours in Figure S1.


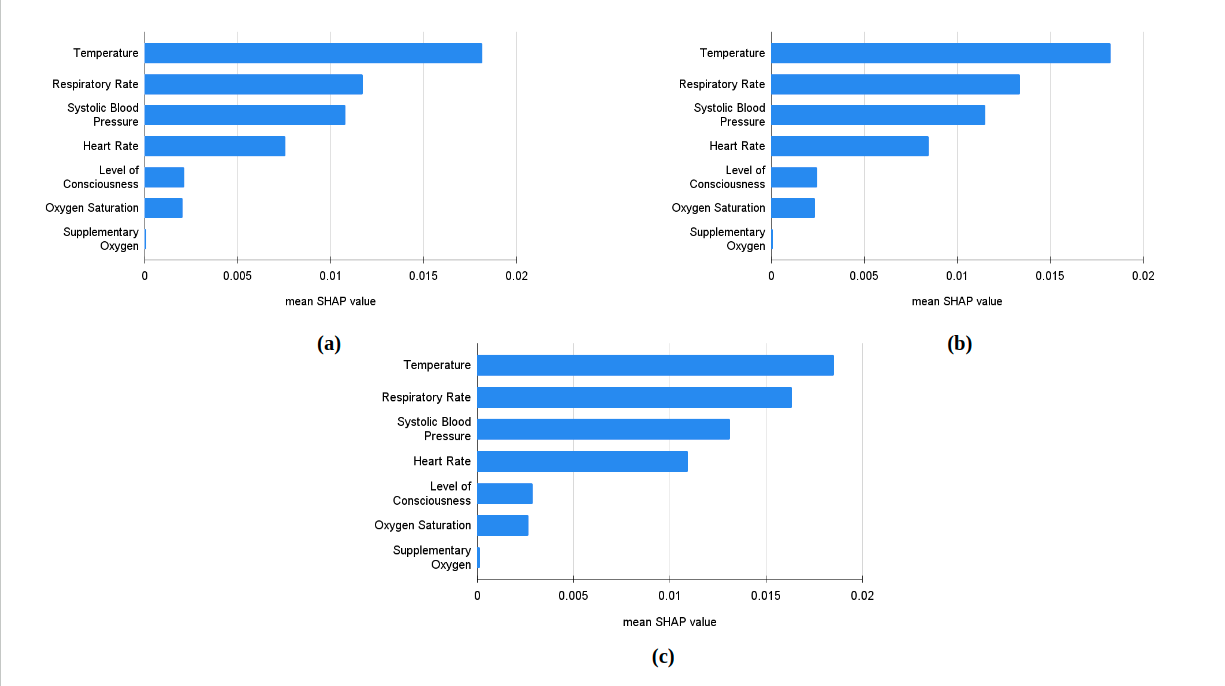


**Figure S1 Feature importance of the XGBoost model.** We present the results of our SHAP analysis for the XGBoost model for deterioration within (a) 6 hours, (b) 12 hours, (c) 36 hours. We provide the mean of the absolute SHAP value for each of the seven input features.

**Hyperparameters**

We report the hyperparameters that we investigated during hyperparameter tuning, as well as the ranges used for each hyperparameter, in Table S2.

**Table S2. Model hyperparameters and ranges for hyperparameter tuning.** We vary hyperparameters for each run by randomly choosing a value within each range for each specified hyperparameter. Hyperparameters not specified are otherwise kept constant.

**Hyperparameter Range**

| **Neural network**  Learning rate | [10-5, 10-3] |
| --- | --- |
| **Logistic Regression**  C | [0.1, 2] |
| **XGBoost**  Column subsample ratio | (0, 1) |
| Max depth | [7, 20] |
| Learning rate | [0.01, 0.5] |
| L1 regularization term | [5, 50] |
| Number of estimators | [5, 20] |
| gamma | [1, 4] |
